# Western Lancet

**Published:** 1842-06-04

**Authors:** 


### THE MEDICAL EXAMINER.

### PHILADELPHIA, JUNE 4, 1842.

*Western Lancet.* We have received the first number of a new medical journal which has been commenced at Cincinnati, edited by Leonidas Moreau Lawson, M. D. It gives evidence of spirit and ability, and, we hope, may be successful.

